# Mortality rates and the causes of death related to diabetes mellitus in Shanghai Songjiang District: an 11-year retrospective analysis of death certificates

**DOI:** 10.1186/s12902-015-0042-1

**Published:** 2015-09-04

**Authors:** Meiying Zhu, Jiang Li, Zhiyuan Li, Wei Luo, Dajun Dai, Scott R. Weaver, Christine Stauber, Ruiyan Luo, Hua Fu

**Affiliations:** Shanghai Songjiang Center for Disease Control and Prevention (CDC), North Xilin Road 1050, Songjiang District, Shanghai, 201620 China; Department of Preventive Medicine, School of Public Health and Key Laboratory of Public Health Safety, Fudan University, Yixueyuan Road 138, PO Box 248, Shanghai, 200032 China; Department of Geosciences, Georgia State University, 24 Peachtree Center Avenue SE, Atlanta, GA 30302 USA; School of Public Health, Georgia State University, 140 Decatur Street, Atlanta, GA 30302 USA

## Abstract

**Background:**

China is one of the countries with the highest prevalence of diabetes in the world. We analysed all the death certificates mentioning diabetes from 2002 to 2012 in Songjiang District of Shanghai to estimate morality rates and examine cause of death patterns.

**Methods:**

Mortality data of 2654 diabetics were collected from the database of local CDC. The data set comprises all causes of death, contributing causes and the underlying cause, thereby the mortality rates of diabetes and its specified complications were analysed.

**Results:**

The leading underlying causes of death were various cardiovascular diseases (CVD), which collectively accounted for about 30 % of the collected death certificates. Diabetes was determined as the underlying cause of death on 28.7 %. The trends in mortality showed that the diabetes related death rate increased about 1.78 fold in the total population during the 11-year period, and the death rate of diabetes and CVD comorbidity increased 2.66 fold. In all the diabetes related deaths, the proportion of people dying of ischaemic heart disease or cerebrovascular disease increased from 18.0 % in 2002 to 30.5 % in 2012. But the proportions attributed directly to diabetes showed a downtrend, from 46.7–22.0 %.

**Conclusions:**

The increasing diabetes related mortality could be chiefly due to the expanding prevalence of CVD, but has nothing to do with diabetes as the underlying cause. Policy makers should pay more attention to primary prevention of diabetes and on the prevention of cardiovascular complications to reduce the burden of diabetes on survival.

**Electronic supplementary material:**

The online version of this article (doi:10.1186/s12902-015-0042-1) contains supplementary material, which is available to authorized users.

## Background

Diabetes is a global epidemic with nearly 350 million cases [1]. According to the data from the World Health Organization (WHO), over 3 million people die worldwide from diabetes and its related complications every year [2]. China is the country with the second highest diabetes rate in the world [3]. The development and expansion of China’s economy over the past decade, has led to dramatic changes in people’s lifestyle and an increase in life expectancy, not only in cities but also in rural areas. But changes in lifestyle and diet associated with an improvements in socioeconomic status may contribute to an increasing diabetes burden in China [4, 5]. A national survey conducted in 2009 estimated age-standardized prevalences of 9.7 and 15.5 % for diabetes and prediabetes, which accounted for 92.4 million and 148.2 million adults, respectively [6]. These estimates are approximately tripled and quintupled, respectively, from earlier estimates from survey data collected in early 1990s [7].

Many people with diabetes do not die from hyperglycaemia itself; the top causes of death are various complications, among which cardiovascular diseases and nephropathy are the most common [8–10]. This results in a difficulty for estimating the burden of diabetes on survival. There are normally two measures estimating the burden of diabetes, *diabetes related death* and *diabetes as the underlying cause of death*. For the *diabetes related death*, underreporting in death certificate is very common worldwide. It was found that only about 40 % diabetes had been recorded on the death certificates of decedents with known diabetes [11–18]. On the other hand, the state of existing routine vital statistics worldwide may omit mention of *diabetes as an underlying cause of death* [19]. The recording cause of death can also be affected by subjective judgement of physicians and different coding systems among countries [20, 21].

In 1980s, China’s Ministry of Health established the vital registration system (MOH-VR) to record the fact and cause of death. However, the accuracy and representativeness of data from the system are biased due to some limitations such as variability in quality control measures and coverage between different areas [22]. Therefore, the Ministry of Health instructed departments of health at the province and county levels to establish the Chinese Disease Surveillance Points System (DSP) from which cause specific mortalities are monitored for a nationally representative sample. In 2013, there were 605 DSP sites nationwide covering over 1 % of the national population [22]. Shanghai Songjiang District is one of the DSP sites since 1990s.

In the present study, a file of coded death certificate data was supplied by the Songjiang Center for Disease Control and Prevention (CDC). The data set comprises all causes of death including the contributing causes and the underlying cause associated with diabetes mellitus between January 2002 and December 2012. From these data, we described the changing patterns of mortalities related to diabetes, and analysed its health burden for the last decade. To the best of our knowledge, this is the first study reporting epidemiological data concerning mortalities related to diabetes in China.

## Methods

Songjiang District is one of the administrative regions of the Shanghai municipality located to the South of the city centre, covering 15 communities including rural and urban areas. According to the data from the government, the population of Songjiang District was approximately 500,000 in 2002. With a natural population growth rate of about 15 per thousand, the registered permanent residents increased to 583,000 in 2012.

Data on mortality status were ascertained through death certificates completed by local physicians, validated by specific staff, and archived (electronic data) with the local CDC. The detailed working procedure for mortality registration and surveillance is described elsewhere [22, 23]. The information in the death certificate includes demographic variables for each decedent, such as sex, date of birth and date of death, all causes of death (related and underlying), as well as some other death status such as death place, diagnostic basis of death, the level of the institute providing diagnosis etc. By considering ethical issues, we used anonymized death registration data with hiding personal identification, so that no individual could be identified. Therefore individual institutional approval was unnecessary. The dataset can be found in Additional file 1.

The present study includes data for all deaths related to diabetes (ICD-10 E10-E14) among the permanent residents, during the 11-year period from 2002 to 2012. The fourth character subdivisions under ICD-10 E10-E14 were used to analyse diabetic complications.

Statistical analysis was performed using the PASW statistics version 18 (SPSS) for Windows. Values of *p* < 0.05 were deemed as statistically significant. Normally distributed continuous clinical data were presented as means ± SD, whereas non-normally distributed continuous data were presented as median and interquartile range (IQR; P25–P75), unless otherwise stated. To test temporal trends, we performed the Spearman rank correlations of mortalities and time. Interval estimations (95 % confidence interval, 95 % CI) were used to analyse statistical significances of proportion differences between 2002 and 2012.

## Results

### Description of total sample

There were 2654 *diabetes related deaths* in the period 2002–2012 (1269 men, 1385 women). This represents 6.6 % (2654/40059) of all deaths in Songjiang District. The age of the deceased ranged between 18 and 101 years, median 76 (IQR: 68 – 82) years. Of these deaths, there were 21 (0.8 %) type 1 diabetics, 1168 (44.0 %) type 2 diabetics, 1462 (55.1 %) cases with unspecified diabetes (ICD-10 E14), and 3 cases that were allocated to other specified diabetes (ICD-10 E13). Overall, diabetes was determined as the underlying cause of death on 28.7 % (762 cases) of the collected death certificates.

For 2332 deceased, the diagnosis of the cause of death was based on medical records, which accounts for 87.9 % of all the involved subjects; 268 (10.1 %) deaths were diagnosed based on pathologic findings; 36 (1.4 %) deaths were determined in surgical operations; and 14 (0.5 %) deaths were determined by executed autopsy. There were 1495 (56.3 %) home deaths and 1045 (39.4 %) deaths occurred in health facilities. The remaining 114 (4.3 %) deaths died on the way to hospital or somewhere else.

Table 1 shows all of the underlying causes of death for the subjects with diabetes from 2002 to 2012. The leading causes of death were cardiovascular diseases, diabetes mellitus, neoplasms, and respiratory diseases. These accounted for 88.4 % of all the causes of death. Women more frequently died from unspecified diabetes, cerebrovascular diseases and injury than men; whereas the cause of death for men was more frequently attributed to tumour, hypertension and respiratory disease than women (*p* < 0.05, Chi-square test).

**Table 1:** Causes of death for diabetics in Songjiang District 2002–2012

	Total no. (%)	Male	Female	*P* value^†^
All causes	2654 (100)	1269 (100)	1385 (100)	-
Diabetes as the underlying cause	762 (28.7)	323 (25.5)	439 (31.7)	<0.01
Type 1 diabetes (E10)	15 (0.6)	7 (0.6)	8 (0.6)	0.93
Type 2 diabetes (E11)	306 (11.5)	134 (10.6)	172 (12.4)	0.13
Unspecified diabetes (E14)	441 (16.6)	182 (14.3)	259 (18.7)	<0.01
Infections (A00-B99)	68 (2.6)	32 (2.5)	36 (2.6)	0.90
Neoplasms (C00-D48)	612 (23.1)	339 (26.7)	273 (19.7)	<0.01
Mental and behavioural disorders (F00-F99)	9 (0.3)	4 (0.3)	5 (0.4)	1.00^‡^
Neuropathies (G00-G99)	15 (0.6)	8 (0.6)	7 (0.5)	0.67
Cardiovascular diseases (CVD) (I00-I99)^a^	793 (29.9)	362 (28.5)	431 (31.1)	0.15
Hypertension (I10-I15)	25 (0.9)	17 (1.3)	8 (0.6)	0.04
Ischaemic heart diseases (I20-I25)	232 (8.7)	112 (8.8)	120 (8.7)	0.88
Cerebrovascular diseases (I60-I69)	522 (19.7)	226 (17.8)	296 (21.4)	0.02
Other CVDs^a^	14 (0.5)	7 (0.6)	7 (0.5)	0.86
Respiratory diseases (J00-J99)	178 (6.7)	113 (8.9)	65 (4.7)	<0.01
Diseases of the digestive system (K25-K93)	63 (2.4)	33 (2.6)	30 (2.2)	0.46
Diseases of the musculoskeletal system and connective tissue (M00-M99)	14 (0.5)	4 (0.3)	10 (0.7)	0.18^‡^
Nephropathy (N00-N19)	16 (0.6)	8 (0.6)	8 (0.6)	0.86
Other disorders of the genitourinary system (N20-N99)	6 (0.2)	4 (0.3)	2 (0.1)	0.43^‡^
External causes (V01-Y98)	111 (4.2)	38 (3.0)	73 (5.3)	<0.01
Others^a^	7 (0.3)	1 (0.1)	6 (0.4)	0.13^‡^

Data are shown as frequencies and percentages

^†^Chi-square test, unless otherwise stated

^‡^Fisher’s exact test

^a^The ICD-10 code I46 is not included in CVD category but in the category of others, since it is not a cause of death referring to CVD

### The burden of diabetic complications

**Fig. 1:**
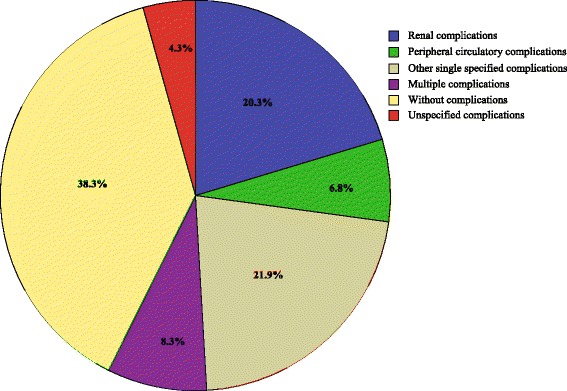
The distribution of diabetes specified complications in the decedents with diabetes as underlying cause of death in 11 years

Of the 2654 *diabetes related deaths*, 1943 (73.2 %) deceased were diagnosed with comorbidity of diabetes and circulatory system diseases (ICD-10 I00–I99, excluding I46); and no statistical difference was shown by gender (*p* = 0.10, Chi-square test). Diabetes specified complications were determined according to the fourth character subdivisions under ICD-10 codes E10–E14. The distribution of complications in the deceased with *diabetes as underlying cause of death* is shown in Fig. 1. In addition to the circa 38 % deceased without complications, the leading causes were renal complications, other specified complications, multiple complications, and peripheral circulatory complications, which collectively accounted for over 45 % of deaths where diabetes was the underlying cause.

### Trends in mortality and proportion during the 11-year period

Of all the *diabetes related deaths*, type 2 diabetes and the unspecified diabetes (ICD-10 E14) accounted for over 97 % of cases in each of every year from 2002 to 2012. However, there were divergent trends: the proportion of unspecified diabetes declined, whereas the proportion of type 2 diabetes presented an ascendant pattern. For more detail please find in Additional file 2: Figure S1.

**Fig. 2:**
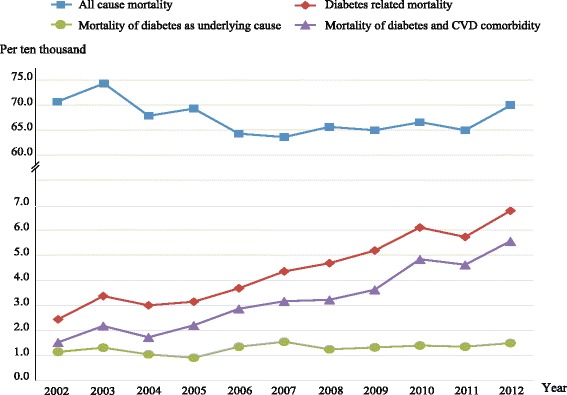
Changing patterns in crude death rates from 2002 to 2012. The denominators are the average total population in Songjiang District for every year

Annual crude mortality rates for all-causes were relatively stable during the 11-year period ranging 63.19 per ten thousand to 74.39 per ten thousand for the Songjiang population (see Additional file 3: Table S1). The diabetes related death rate was 2.44 per ten thousand in 2002, increasing to 6.78 per ten thousand in 2012. The death rates of diabetes and cardiovascular diseases comorbidity exhibited the same rising trend, from 1.52 per ten thousand in 2002 to 5.55 per ten thousand in 2012. The mortalities of *diabetes as the underlying cause* rose slightly from 1.14 per ten thousand in 2002 to 1.49 per ten thousand in 2012. These rates are shown in Fig. 2. The Spearman’s rho were −0.24 (*p* = 0.31), 0.89 (*p* < 0.01), 0.49 (*p* = 0.04), and 0.89 (*p* < 0.01) for the correlations between all-cause mortality and the year of death, between diabetes related death rate and the year of death, between the mortality of diabetes as the underlying cause and the year of death, between the death rate of diabetes and CVD comorbidity and the year of death, respectively.

**Fig. 3:**
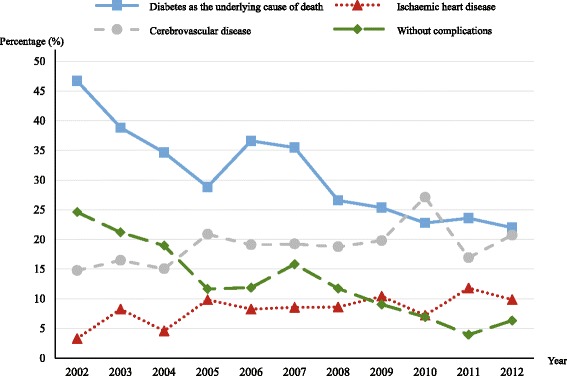
Changing patterns in proportions of the underlying cause of death for diabetes, cardiovascular diseases, and diabetes without complications. The denominators are the number of diabetes related deaths for every year

Of all *diabetes related deaths*, the proportions attributed directly to diabetes as the underlying cause decreased from 46.7 % in 2002 to 22.0 % in 2012 (95 % CI of the rate difference ranged from 15.0–34.5 %). The corresponding Spearman’s rho = −0.92 (*p* < 0.01). The proportions of deceased without complications followed a similar downtrend from 24.6 in 2002 to 6.3 % in 2012 (95 % CI of the rate difference ranged from 10.3–26.3 %). The Spearman’s rho = −0.93 (*p* < 0.01). In comparison, the proportions of deceased diabetics who died from renal complications varied within a narrow range, from 6.6 % in 2002 to 5.1 % in 2012 (95 % CI of the rate difference ranged from −3.4–6.4 %). The Spearman’s rho = −0.45 (*p* = 0.17). The proportions of deceased diabetic due to cardiovascular diseases showed an ascendant trend, of which ischaemic heart diseases as underlying cause of death increased from 3.3 % in 2002 to 9.8 % in 2012 (95 % CI of the rate difference ranged from −10.9–2.3 %), and cerebrovascular diseases increased from 14.8 % in 2002 to 20.7 % in 2012 (95 % CI of the rate difference ranged from −13.4–1.5 %). The values of Spearman’s rho were 0.70 (*p* = 0.02) and 0.60 (*p* = 0.05), respectively. These changing patterns are shown in Fig. 3.

## Discussion

Trends in mortalities tend to reflect the level of economic development and the changing pattern of health services of a country or region [24, 25]. In the present study, it showed an increased trends in diabetes related mortality with growing proportions of CVD prevalence and mortality in a Chinese population. In the first decade of the 21^st^ century, China has been one of the most rapidly developing countries in the world with an increase in life expectancy and changes in lifestyle [4]. The crude death rates for China, including for Shanghai [26] and Songjiang have varied only slightly during this decade, but the diabetes mortality (*diabetes related death*) increased about 1.78 fold (see Fig. 2) in Songjiang District. Similarly, the prevalence of diabetes has increased dramatically during a similar period in China, from 5.5 % in 2001 [27] to 11.6 % in 2010[28]. This reveals that the increased diabetics should be owed to the growth of incidence, which should be faster than the growth of mortality. Certainly, the development of medical techniques should help to detect more and more individuals with undiagnosed diabetes since last decades, which could led to a pseudo increase of incidence. In spite of the striking increase of *diabetes related death* (by 178 %), the mortality of *diabetes as the underlying cause* increased only 30 %. In addition, the proportion of *diabetes as the underlying cause* reduced significantly, of which especially the proportion of subject without complications (Fig. 3). This may reflect improvement of health service like effective treatment of hyperglycaemia and efforts to prevent diabetic complications (not counting CVD) by local health care systems. On the other hand, it suggests that the central and local governments should pay more attention to primary preventions aimed at lowering the incidence and ultimately to reduce the burden of diabetes. Moreover, the descending proportion of unspecified type may be attributable to improvements in proper diagnosis by clinicians. That is, the types of diabetes for a large amount of decedents had been already clarified and/or recorded before death. This is in conformity with the progress of local health service (Data from “Statistic Yearbook of Songjiang” 2004 – 2013,published by local government).

People with type 2 diabetes have an increased risk of developing CVD. In the present study, 73.6 % of deceased diabetics had suffered from CVD, and about 30 % died directly of CVD, which is higher than India (13.7 %) [29] and Taiwan (19.8 %) [30], and is lower than the western countries (49.4 % for the U.S. and 49.1 % for U.K.) [31]. But this comparison is not totally appropriate because it can be biased by many factors including the difference of coding system among countries, the habits of recording physicians, the subjective judgement of them, and so on [20, 21]. We used continuous data to describe the variation of mortality of CVD, which is more informative than a static observation and helpful to assess the CVD burden. The changing pattern during the 11-year period manifests that the increasing deaths associated with diabetes should chiefly be owed to cardiovascular diseases but have nothing to do with *diabetes as the underlying cause*, that is, other diabetic complications and diabetes without complications. As shown in Fig. 2, the curve of death rate for people with diabetes and CVD comorbidity shows nearly parallel to the curve of diabetes related mortality. In fact, the mortality for people with comorbidity increases faster than the mortality for all diabetics, that is, the proportion of deceased diabetics with CVD comorbidity increased from 62–82 % during the 11 years. However, the mortality rate for people with other complications varied very slightly; even the proportion of deaths without complications decreased more than three fold in the same period.

Mortality statistics are an important source of health information for analysing the burden of diseases and are often used to assign priorities in health policy [2]. In China, there are two sets of mortality registration systems, the MOH-VR and the DSP. In general, the DSP provides more representative and more accurate data than the MOH-VR, especially in rural areas [22]. Songjiang District is one of the national DSP sites since the last decades, where the non-agricultural population accounted for 46 % in 2003 and 83 % in 2012 (Data from “Statistic Yearbook of Songjiang” 2004 – 2013). Considering that Songjiang locates in the most developed area in China, the number of hospital beds per thousand people was 6.5 in 2003 and 8.0 in 2012, that is, the local health service are quantitatively and qualitatively in the forefront of the country [26], thus the accuracy of the data should be in a higher level comparing with other areas of the country.

In general, estimation of diabetes mortality is challenging due to various reasons. For example, these challenges stem from people with diabetes most frequently dying of cardiovascular diseases or renal failure, and the physicians who record and determine the causes of death may miss and underestimate the contributions of diabetes on death [11–19, 32]. In the present study, the death certification in Songjiang listed up to seven causes contributing to death, of which the underlying causes were determined by certified doctors. We collected all records mentioning diabetes in the certificates to assess contributions of diabetes on death; thus the estimate we calculated could be very similar to a mortality statistic for the people with diabetes.

Although we are confident of the higher quality of data, data from death certificates have potential to distort mortality rates because of the ineluctability of incorrect reporting. One possible explanation for the steep increase in *diabetes related deaths* could be increased awareness of the disorder. Since the Center for Non-communicable Disease of the Chinese Center for Disease Control and Prevention (China CDC) was established in 2002, surveillance on chronic diseases, such as hypertension and diabetes etc., was gradually highlighted by local CDCs. Therefore, an underestimation of mortality should be taken into account, especially in the earlier stage of the investigation. Furthermore, the International Statistical Classification of Diseases and Related Health Problems (known as ICD) facilitates mortality statistics by standardizing causes of death [33], and also request the capacities of certifying doctors in the correct ICD procedures [34]. But even in developed countries there was often substantial use of ICD codes for unknown and ill-defined causes [33]. Also ill-defined causes (ICD-10 codes R00-R99) existed in our data (230 cases for the contributing causes, but none was in the underlying cause), that would also affect the validity of our estimates. Death certificate is one of the most important sources of data regarding people health, but the data quality is often concerned. There were few studies concerning the accuracy of recorded cause of death of diabetic decedents in China. Therefore, assessment and further improvement of death certificate should become a key emphasis in work of the health care system in the future, which would help us to correctly understand the burden of diabetes on survival [35].

Another limitation relates to the representativeness of local population. Due to China’s household registration system, only permanent residents were recorded by local CDCs. People who lived in Songjiang but were registered in other regions were not included in the present study. Because the majority of these people were young adults who worked in Songjiang, the diabetes related mortalities for the total population would be smaller than the data in the present study. Moreover, though the data of the study cannot represent the characteristics of entire Chinese population, it should be also meaningful and suggestive for policymakers. Since Shanghai is one of the most developed area of China and has a very higher proportion (>20 %) of old people (60 years or older, permanent residents) [36]. This is and will be a similar situation in other areas of China.

The present survey provides only information restricted in deceased diabetics, therefore the results should be explained carefully. For example, although the proportions of those who died from CVD rose rapidly from 2002 to 2012, we cannot attribute the increase of CVD mortality only to the prevalence of diabetes. Because we did not collect mortality data of people who did not have diabetes, we do not know the situation of CVD mortality for them [37]. In contrast, from the changing proportions it could be clearly determined that CVD contributed more and more on diabetes deaths from 2002 to 2012.

## Conclusions

There was an increase in *diabetes related mortality* over the past decade. The increased mortality appears mostly attributed to the prevalence of cardiovascular diseases. Therefore, policy makers should pay more attentions to diabetes primary prevention to reduce the incidence rate while also placing a priority on the prevention of cardiovascular diseases to reduce the burden of diabetes on survival.

## Additional files

Additional file 1:
**Dataset.** This Excel dataset was used for all the analysis of the present study. (XLS 602 kb) Additional file 2: Figure S1. The Proportion of diabetes types in the diabetes related deaths from 2002 to 2012. The blocks indicate the proportions of type 2 diabetes, unspecified type, and other specified types (including type 1 diabetes) in each year, respectively. (PDF 154 kb) Additional file 3: Table S1. Annual number of deaths according to different sub-groups from 2002 to 2012. (PDF 43 kb)

## Abbreviations

CVD: Cardiovascular disease
WHO: World Health Organization
MOH-VR: Ministry of Health –vital system
DSP: Disease Surveillance Points System
CDC: Center for Disease Control and Prevention
IQR: Interquartile range
ICD: International Statistical Classification of Diseases
