# Supplementary figures and images for: Mortality rates and the causes of death related to diabetes mellitus in Shanghai Songjiang District: an 11-year retrospective analysis of death certificates
Source: BMC Endocr Disord. 2015 Sep 4;15:45. doi: 10.1186/s12902-015-0042-1 (PMC4559917; doi:10.1186/s12902-015-0042-1)

■ Type 2 diabetes    ■ Unspecified diabetes    ■ Other specified diabetes

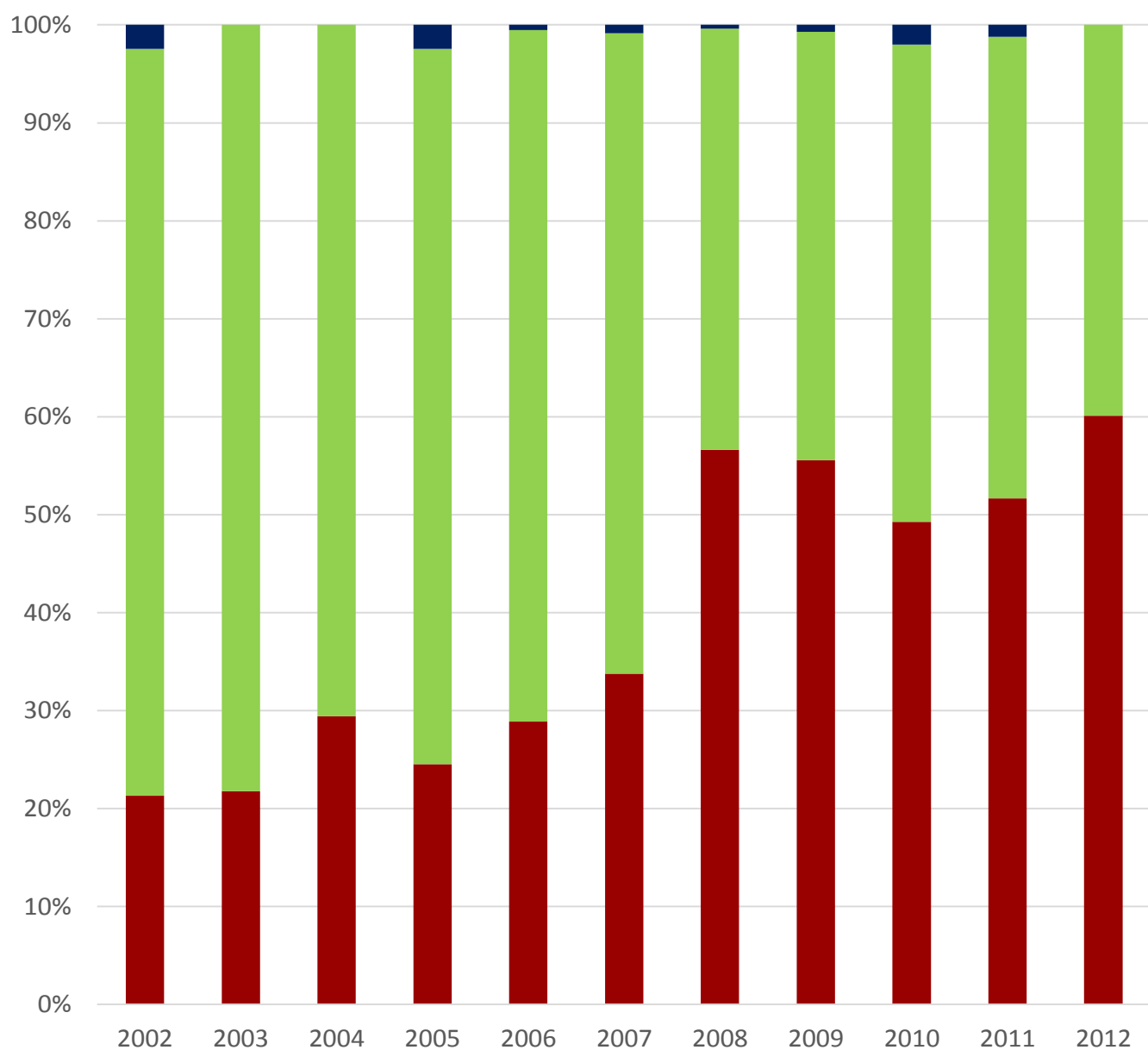

Supplement: Additional file 2: Figure S1. — The Proportion of diabetes types in the diabetes related deaths from 2002 to 2012. The blocks indicate the proportions of type 2 diabetes, unspecified type, and other specified types (including type 1 diabetes) in each year, respectively. (PDF 154 kb) [file 12902_2015_42_MOESM2_ESM.pdf]
