# Supplementary material for: Mortality rates and the causes of death related to diabetes mellitus in Shanghai Songjiang District: an 11-year retrospective analysis of death certificates
Source: BMC Endocr Disord. 2015 Sep 4;15:45. doi: 10.1186/s12902-015-0042-1 (PMC4559917; doi:10.1186/s12902-015-0042-1)
Supplement: Additional file 3: Table S1. — Annual number of deaths according to different sub-groups from 2002 to 2012. (PDF 43 kb) [file 12902_2015_42_MOESM3_ESM.pdf]

Table. Annual number of deaths according to different sub-groups from 2002 to 2012

Year	Total population	All causes of death	Deaths related to diabetes	Deaths with diabetes and CVD comorbidity	Diabetes as the underlying cause of death	Ischaemic heart disease*	Cerebrovascular diseases†	Renal complications‡	Without complications#
2002	500579	3570	122	76	57	4	18	8	30
2003	505016	3757	170	110	66	14	28	10	36
2004	510612	3437	153	88	53	7	23	8	29
2005	518260	3605	163	112	47	16	34	9	19
2006	527141	3404	194	147	71	16	37	19	23
2007	537428	3396	234	169	83	20	45	14	37
2008	546576	3602	256	176	68	22	48	12	30
2009	554941	3607	288	201	73	30	57	16	26
2010	567737	3820	347	275	79	25	94	22	24
2011	577609	3768	331	265	78	39	56	17	13
2012	583981	4094	396	324	87	39	82	20	25
Total	-	40060	2654	1943	762	232	522	155	292

* The number of death of diabetics with ischaemic heart disease (ICD-10 codes I20-I25) as underlying cause of death.

†The number of death of diabetics with cerebrovascular diseases (ICD-10 codes I60-I69) as underlying cause of death.

‡The number of death of diabetic renal complications (ICD-10 codes E10-E14 tailing the fourth character of 2) as underlying cause of death.

#The number of death of diabetics without complications (ICD-10 codes E10-E14 tailing the fourth character of 9) as underlying cause of death.
